# The New Metals

**Published:** 1870-02

**Authors:** 


The New Metals.—When we use the expression "*new metals*," we mean those the knowledge of which has come to mankind in recent times, in contradistinction to those that were known in the early periods of human history.

Some of these *new metals* have been familiar for centuries, and some are the discoveries of yesterday. Our purpose is to mention them all.

Metals necessarily stand in the first rank of all the materials used by man. Their tenacity, their malleability and ductility, enabled mankind from the very first, to convert them into implements of the chase, fishery, agriculture and war.

The first thought of the early races, in the effort to supply their most pressing needs. was to make sharp weapons of stone equal to the task of cutting anything as they wished to shape it; but in applying metals to the same use they realized an immense progress.

One circumstance that retarded their application to the arts was, that they are rarely found in their natural condition. In ancient times, gold and silver only offered this advantage, which is the result of their inalterability, and the latter property it is that won for them the designation of *precious* metals.

Mercury and copper were also found sometimes in their native state; but all the other metals, disguised as they were in clayey or stony forms, demanded a greater or less degree of labor to get them out. The commencement of these toils is lost in the night of time.

Toward the close of the last century, which marked the first great revival of chemistry, we knew but *nine* metals which were, so to speak, classic, viz—gold, silver, mercury, copper, iron, lead, tin, antimony, and bismuth; to which were soon added, arsenic, nickel, cobalt, manganese, zinc, and cadmium. At this point, indeed, science seemed to enter upon a limitless succession of novelties, of which the generally and certainly ascertained enumeration includes some *fifty-one* metals in all, as follows:

Gold, silver, copper, platinum, lead, mercury, iron, tin, antimony, bismuth, aluminum, barium, cadmium, calcium, cerium, chrome, cobalt, cæsium, didymium, erbium, glucinium, iridium, ilmenium, lantanium, lithium, molybdenium, nickel, osmium, pelopium, rhodium, ruthenium, strontium, terbium, thorium, tungsten, vanadium, zinc, magnesium, manganese, niobium, palladium, potassium, rubidium, sodium, tantalum, thalium, titanium, uranium, yttrium, zirconium, arsenic.

Nothing would be more curious, perhaps, than a general review of the benefits that human industry and skill have succeeded in drawing from all this metallic wealth, the most of which is due to the 19th century. How some are remarkable for rigidity, tenacity, malleability, ductility, or fusibility, and others by their inalterability, or infusibility, each property which, in certain cases, becomes a defect, bestowing a peculiar quality for other applications, or, at least, amounting to a facility for manufacture.

Since the day when it was suspected that every earthy substance represented the oxide of a metal, a strenuous attempt has been made to reduce these earthy deposits to their metallic condition. Success has been obtained in the laboratories by the use of the voltaic battery; and this fact once demonstrated, recourse was had to the ordinary processes of metallurgy, so as to produce the same effect upon a larger scale. It was thus that a whole class of metals previously unknown, was obtained, such as potassium, sodium, calcium, magnesium, barium, strontium, etc. When operating with the battery, it was very hard to obtain even a simple globule, while upon substituting the action of fire, chemists were enabled to volatilize the same metals, to distil them, so to speak, and to condense them in receptacles prepared for the purpose.

The question was then quickly asked, "What is the use of these metals which, upon contact with water or the atmosphere, immediately return to their earthy condition, unless it be simply as matters of curiosity, or for the sake of producing a display of fireworks on a small scale?" But in all the sciences, every new fact possesses a relation to others, unforeseen at first, but which sooner or later, bears fruit. Potassium and sodium, for instance, served in the first place to disengage silicium and borium, and now, sodium, extracted from carbonate of soda, serves to prepare aluminum for industrial uses.

The metals of the *platinum* family are ranged in a different category. These being inoxydables, present themselves in the metallic condition naturally; but they can not be melted for the purposes of the workman. They are found in grains frequently as hard as tempered steel, and, in order to separate them, the *inverse method* has had to be employed; that is to say, it has been found necessary to dissolve them with acids, and then to bring them back again, separately, to the metallic condition. It was after this fashion that palladium, platinum, rhodium, iridium, osmium, etc., were discovered. Palladium alone could be melted in the first instance; but platinum might be divided into very thin scales, and drawn out into the finest threads without melting. Now, all of them are melted excepting osmium, which bears great analogy to carbon.

It would be difficult to enumerate all the advantages that the researches of the laboratory and certain manufacturing operations have derived from the use of potassium vessels. To them is, in a great measure, due the precision of modern analysis, and they have served almost exclusively, until the present day, for the concentration of sulphuric acid, which, to some degree forms the starting point for all kinds of chemical transformations.—*New York Mercantile Journal.*

